# Identifying a Ferroptosis-Related Gene Signature for Predicting Biochemical Recurrence of Prostate Cancer

**DOI:** 10.3389/fcell.2021.666025

**Published:** 2021-10-29

**Authors:** Zhengtong Lv, Jianlong Wang, Xuan Wang, Miao Mo, Guyu Tang, Haozhe Xu, Jianye Wang, Yuan Li, Ming Liu

**Affiliations:** ^1^Department of Urology, Beijing Hospital, National Center of Gerontology, Institute of Geriatric Medicine, Chinese Academy of Medical Sciences, Beijing, China; ^2^Graduate School of Peking Union Medical College and Chinese Academy of Medical Sciences, Beijing, China; ^3^Department of Urology, Xiangya Hospital, Central South University, Changsha, China; ^4^Department of Urology, The Second Xiangya Hospital, Central South University, Changsha, China

**Keywords:** prostate cancer, TCGA, MSKCC, ferroptosis, prognosis, biochemical recurrence

## Abstract

Ferroptosis induced by lipid peroxidation is closely related to cancer biology. Prostate cancer (PCa) is not only a malignant tumor but also a lipid metabolic disease. Previous studies have identified ferroptosis as an important pathophysiological pathway in PCa development and treatment, but its role in the prognosis of PCa is less well known. In this study, we constructed a nine-ferroptosis-related gene risk model that demonstrated strong prognostic and therapeutic predictive power. The higher risk score calculated by the model was significantly associated with a higher ferroptosis potential index, higher Ki67 expression, higher immune infiltration, higher probability of biochemical recurrence, worse clinicopathological characteristics, and worse response to chemotherapy and antiandrogen therapy in PCa. The mechanisms identified by the gene set enrichment analysis suggested that this signature can accurately distinguish high- and low-risk populations, which is possibly closely related to variations in steroid hormone secretion, regulation of endocrine processes, positive regulation of humoral immune response, and androgen response. Results of this study were confirmed in two independent PCa cohorts, namely, The Cancer Genome Atlas cohort and the MSK-IMPACT Clinical Sequencing Cohort, which contributed to the body of scientific evidence for the prediction of biochemical recurrence in patients with PCa. In addition, as the main components of this signature, the effects of the *AIFM2* and *NFS1* genes on ferroptosis were evaluated and verified by *in vivo* and *in vitro* experiments, respectively. The above findings provided new insights and presented potential clinical applications of ferroptosis in PCa.

## Introduction

The latest cancer statistics show that prostate cancer (PCa) has surpassed lung cancer, as it becomes the malignant tumor with the highest incidence in men and ranks second to lung cancer in terms of mortality rate (Siegel et al., 2020). Radical prostatectomy, external-beam radiation therapy, and brachytherapy are recommended interventions for localized PCa (Mottet et al., 2020). Although these radical treatments can control the disease chronically, several patients (20–25%) still experienced biochemical recurrence (BCR) during the follow-up period (Pound et al., 1999; Simmons et al., 2007; Van den Broeck et al., 2019). Patients with BCR who did not receive secondary therapy will develop clinical progression within 5–8 years, and 32–45% will die of the disease within 15 years (Brockman et al., 2015). Therefore, identifying new biomarkers is crucial to predict high-risk PCa patients with high BCR risk.

Ferroptosis is a new kind of regulated cell death (RCD) and is different from apoptosis, necrosis, and autophagy in terms of morphology, biochemistry, and genetics (Stockwell et al., 2017). In the last 8 years, the complex relationship between ferroptosis and cancer has incited widespread concern (Tang D. et al., 2020). Changes in iron metabolism not only promote the growth and proliferation of tumor cells by increasing iron reserves (Manz et al., 2016) but also induce excessive iron concentration that can cause lipid peroxidation in the tumor cell membrane and lead to ferroptosis. Over the past 5 years, basic and clinical researchers have shown a growing interest in the role of ferroptosis in the pathogenesis of cancer (Chen et al., 2021). At present, triggering ferroptosis, as a new method of treating cancer, has received high expectations and has been an area of active research (Hassannia et al., 2019; Liang et al., 2019).

Numerous studies have demonstrated the critical role of ferroptosis in the development and treatment of PCa. For example, enzalutamide was found to induce lipid uptake and remodeling, which in turn induces ferroptosis hypersensitivity (Tousignant et al., 2020). Flubendazole can induce potent antitumor effects by targeting P53 and promoting ferroptosis in castration-resistant PCa (CRPC) (Zhou et al., 2021). Erastin is a classical inducer of ferroptosis and can suppress the transcriptional activities of both the full-length and splice variants of androgen receptors (AR) in CRPC (Yang Y. et al., 2021). Qin et al. (2021) designed and synthesized an isothiocyanate-containing hybrid AR antagonist that can efficiently downregulate AR/AR splice variant and induce ferroptosis in CRPC cells combined with glutathione (GSH) synthesis inhibitor. There was a suggestion that the induction of ferroptosis is a new therapeutic strategy for advanced PCa, which can be used as a monotherapy or as a combination therapy with second-generation antiandrogens (Ghoochani et al., 2021). However, the effect of ferroptosis on the prognosis of PCa has rarely been reported.

This study identified ferroptosis-related genes associated with long-term BCR of PCa and finally constructed a prognostic signature based on nine ferroptosis-related genes, which can accurately identify patients with high-risk PCa. The findings provide evidence on the key role of ferroptosis in PCa development.

## Materials and Methods

### Data Acquisition

This study included two independent PCa cohorts. Data of the PCa patient cohort were downloaded from The Cancer Genome Atlas (TCGA) database and used as the training set (2021.03.01), which included 483 patients with comprehensive transcriptome (FPKM standardized data) and clinical information^1^. Data of the MSK-IMPACT Clinical Sequencing Cohort (MSKCC) PCa cohort were obtained from the Cbioportal database^2^ and used as the validation set, which included 138 patients with complete expression profile (normalized log2 mRNA expression data) and clinicopathological information. In addition, several Gene Expression Omnibus (GEO) datasets were selected to verify our results, including GSE54460, GSE70769, GSE104749, GSE88808, GSE70768, GSE69223, GSE68555, GSE55945, GSE46602, GSE38241, GSE35988, GSE32571, GSE32448, GSE6919, and GSE3325^3^. The 84 ferroptosis-related genes were taken from a previous study (Liang et al., 2020) and the ‘‘WP_FERROPTOSIS’’ gene set, which was obtained from the Molecular Signatures Database^4^. The clinicopathological features of the two cohorts are summarized in Table 1.

**TABLE 1:** The basic characteristics of the two cohorts of PCa.

Clinical characteristics	Classification	TCGA (*n* = 472)	MSKCC (*n* = 138)
Age		60.87 ± 6.80	58.03 ± 6.63
PSA		10.86 ± 11.67	12.14 ± 44.08
Gleason score	6	44 (9.32%)	41 (29.71%)
	7	237 (50.21%)	77 (55.80%)
	8	57 (12.08%)	10 (7.25%)
	9	131 (27.75%)	10 (7.25%)
	10	3 (0.64%)	0 (0%)
WHO ISUP	1	44 (9.32%)	41 (29.71%)
	2	142 (30.08%)	53 (38.41%)
	3	95 (20.13%)	24 (17.39%)
	4	57 (12.08%)	10 (7.25%)
	5	134 (28.39%)	10 (7.25%)
T stage	T2a	12 (2.54%)	8 (5.80%)
	T2b	10 (2.12%)	48 (34.78%)
	T2c	158 (33.47%)	29 (21.01%)
	T3a	152 (32.20%)	29 (21.01%)
	T3b	130 (27.54%)	13 (9.42%)
	T3c	0 (0.00%)	4 (2.90%)
	T4	10 (2.12%)	7 (5.07%)
Biochemical recurrence	Yes	87 (18.43%)	35 (25.36%)
	No	385 (81.57%)	103 (74.64%)

*PCa, prostate cancer; TCGA, The Cancer Genome Atlas; MSKCC, MSK-IMPACT Clinical Sequencing Cohort; PSA, prostate-specific antigen.*

### Construction of the Ferroptosis-Related Prognostic Signature

The risk prognostic model was constructed based on the TCGA cohort. The “Limma” package was used to obtain differentially expressed ferroptosis-related genes between PCa tissues and normal tissues, with a false discovery rate (FDR) of <0.05 as the boundary. Univariate Cox regression analysis was employed to identify the ferroptosis-related genes associated with the BCR of PCa. The genes contained in the intersection of the above two analyses were used as the core genes to construct the protein interaction network based on the STRING database^5^ and the expression correlation network based on the expressions of these core genes. Lasso regression was employed to avoid overfitting of the final prediction model. The calculation formula of the model is as follows: $Riskscore=\sum_{m=1}^{n}Coef_{m}\times Exp_{m}$, where Coef_m_ is the risk coefficient and Exp_m_ is the relative mRNA expression of each ferroptosis-related gene. The median risk score was used as a cutoff to distinguish patients with high- and low-risk PCa.

### Evaluation and Validation of the Prognostic Signature

The TCGA and MSKCC cohorts were used to evaluate and verify the prognosis model separately, and the same statistical methods were applied in both cohorts. Log-rank and Kaplan–Meier (K–M) tests were used to visualize the difference in the biochemical relapse-free survival (bRFS) between the two risk groups. The sensitivity and specificity of survival prediction were tested by the receiver operating characteristic (ROC) analysis. An area under the ROC curve (AUC) was calculated as an index of the prediction accuracy, and “vegan” and “stats” packages were used for the principal component analysis (PCA) to explore the distribution among groups. Univariate and multivariate Cox regression analyses were used to investigate whether the risk score can be used as an independent prognostic factor in PCa. The correlation between the risk score and other clinicopathological features was shown by a heat map.

### Gene Set Enrichment Analysis

Hallmark and Gene Ontology (GO) gene sets were downloaded for the gene set enrichment analysis (GSEA) to determine which gene sets were significantly different between the high- and low-risk groups. A total of 1,000 gene set permutations were performed to finally obtain the normalized enrichment score, normalized *p*-value, and FDR. The ferroptosis potential index (FPI) was calculated according to the methods of Liu et al. (2020c) by subtracting the enrichment score of the negative- from the positive-core machine components through single-sample GSEA (ssGSEA) by using the “GSVA” package.

### Immune Infiltration and Tumor Microenvironment

Single-sample GSEA was also used to quantify the immune infiltration level (Rooney et al., 2015). The annotated gene set file was derived from the study of Liang et al. (2020). Finally, the enrichment levels of 16 immune cells and 13 immune-related pathways in each PCa sample were quantified, and the results were expressed by box plots. Moreover, we predicted the TME of PCa by using the “ESTIMATE” package to calculate the immune/stromal/ESTIM/tumor purity score (Yoshihara et al., 2013).

### Prediction of Antiandrogenic Therapy and Immunotherapy Responses

The response of each patient with PCa in both cohorts to bicalutamide and docetaxel was obtained from the Genomics of Drug Sensitivity in Cancer^6^ using the “pRRophetic” R package (Geeleher et al., 2014). Half-maximal inhibitory concentration (IC50) was used to measure the response of tumor cells to drugs.

### Cell Culture

Human prostate and PCa cell lines (PWR1E and DU145) were obtained from the American Type Culture Collection (Manassas, VA, United States) and the Shanghai Institute of Biochemistry and Cell Biology, Chinese Academy of Sciences (Shanghai, China), which were cultured in keratinocyte serum-free medium containing 50 μg/ml bovine pituitary extract and 5 ng/ml epidermal growth factor for PWR1E and RPMI-1640 medium supplemented with 2 mM L-Glutamine, 10% fetal bovine serum, 1% penicillin/streptomycin for DU145. These cells were grown at 37°C with 5% CO_2_ in a humidified incubator. The lentiviral vector carrying AIFM2 shRNA or NFS1 shRNA and the corresponding control shRNA were synthesized and purchased from GenePharma and GenScript (China), respectively.

### Cell Viability Assay

Cell viability was assessed using the MTT assay to evaluate the effects of different concentrations of erastin on cell viability. PWR1E or DU145 cell lines at 2 × 10^3^ cells/well were incubated in 96-well plates for 24 h. An MTT solution (5 mg/ml) was cultured for 4 h. The cells were then treated with 150 μl of dimethylsulfoxide. Cell viability was determined by enzyme-linked immunosorbent assay at 570 nm.

### Ferroptosis-Related Analysis

Various cell biological methods were used to evaluate ferroptosis. Lipid peroxidation was assessed using the BODIPY^TM^ 581/591 C11 (D3861, Thermo Fisher Scientific), and oxidation of the polyunsaturated butadienyl portion of the dye results in the shifting of the fluorescence emission peak from ∼590 (red) to ∼510 nm (green). PWR1E or DU145 cell lines were incubated with 10 μM erastin for 24 h. Then 1 μg/ml of Hoechst 33342 and 1 μm of BODIPY^TM^ 581/591 C11 were added to the culture medium for living cell imaging at the last hour of incubation. Ferroptosis-related indexes, including the levels of Fe^2+^ release, malondialdehyde (MDA), reactive oxygen species (ROS), and GSH, were determined using the Iron Assay Kit (ab83366, Abcam, United Kingdom), lipid peroxidation (MDA) assay (ab118970, Abcam), DCF ROS/RNS Assay (ab238535, Abcam), and GSH/GSSG Ratio Detection Assay Kit II (ab205811, Abcam), respectively.

### Real-Time Quantitative Polymerase Chain Reaction Analysis and Western Blotting

Total RNA was extracted from the cells using Trizol reagent (Invitrogen, Waltham, MA, United States). Approximately 500 ng of RNA was used for the reverse transcription reaction with PrimeScript RT Master Mix [Takara Biotechnology (Dalian) Co., Ltd., Japan]. Then, with GAPDH as the internal control, real-time quantitative polymerase chain reaction (RT-qPCR) was performed using Premix Ex Taq^TM^ II [Takara Biotechnology (Dalian) Co., Ltd.] with the Roche Light Cycler 480 Real-Time PCR system. The sequence of primers is as follows: AIFM2 forward: TTACAAGCCAGAGACTGACCAA, reverse: ACAAGGCCTGTCACTGAAGAG; NFS1 forward: CA CTCCCGGACACATGCTTAT, reverse: TGTCTGGGTGGTGAT CAAGTG; GAPDH forward: ATCATCAGCAATGCCTCCTG, reverse: ATGGACTGTGGTCATGAGTC.

Protein samples (50 μg) were separated by sodium dodecyl sulfate–polyacrylamide gel electrophoresis in 4–12% gel and transferred to nitrocellulose membranes for reaction with antibodies against target genes. Secondary antibodies, i.e., horseradish peroxidase-conjugated rabbit anti-goat and rabbit anti-mouse IgG, were detected by using SuperSignal Chemiluminescent substrate (Pierce Biotechnology, Rockford, IL, United States).

### Immunohistochemistry

Tumors and adjacent normal tissues of 52 patients with PCa who underwent radical prostatectomy at the Beijing Hospital, Xiangya Hospital, and the Second Xiangya Hospital were examined by immunohistochemistry (IHC). Sections were incubated with anti-AIFM2 and anti-NFS1 (1:100 dilution). The sections were scored for staining intensity according to the following scale: negative, no staining; weak, weak staining (light yellow); moderate, moderate staining (yellowish brown); and strong, strong staining (brown). The experiments were reviewed and approved by the Ethics Committee of the Beijing Hospital (2018BJYYEC-085-03). The patients provided their written informed consent to participate in this study.

### Colony Formation Assay

Prostate cancer cells in the logarithmic growth stage were put into six-well plates. The culture continued for 1–3 weeks, during which the solution was changed every 3 days, and the cell status was observed every day until the number of cells in most single clones was greater than 50. Then the cells were cleaned with phosphate-buffered saline buffer, made in methanol approximately 30 min, and stained with crystal violet dye at a dose of 1%. Thereafter, the number of colonies was calculated.

### Tumorigenicity in Nude Mice

All animal experiments were approved by the Institutional Ethics Committee of Xiangya Hospital, Central South University. A subcutaneous xenograft model was established by subcutaneously injecting male nude mice with 1 × 10^6^ DU145 cells on the right side. The tumor volume was measured with calipers and repeatedly measured every 7 days (length × width^2^)/2. At 28 days following implantation, the mice were euthanized by cervical dislocation. Then, the xenografts were removed, fixed, weighed, photographed, and preserved.

### Statistical Analysis

All data sorting and analyses were completed by the R 3.6.1 software. For continuous variables with normal distribution and homogeneity of variance, an independent sample *t*-test was used; otherwise, Wilcoxon rank-sum test was selected. Pearson correlation coefficient test was used to analyze the correlation. A value of *p* < 0.05 was considered significant. Based on the results of the multivariate Cox proportional hazards analysis, a nomogram was developed to predict 1-, 3-, and 5-year bRFS rates. To evaluate the prediction performance of the nomogram, the consistency index (C index), ROC, and calibration curve were used to evaluate the nomogram.

## Results

### Development of the Prognostic Ferroptosis-Related Signature of Patients With Prostate Cancer

**FIGURE 1:**
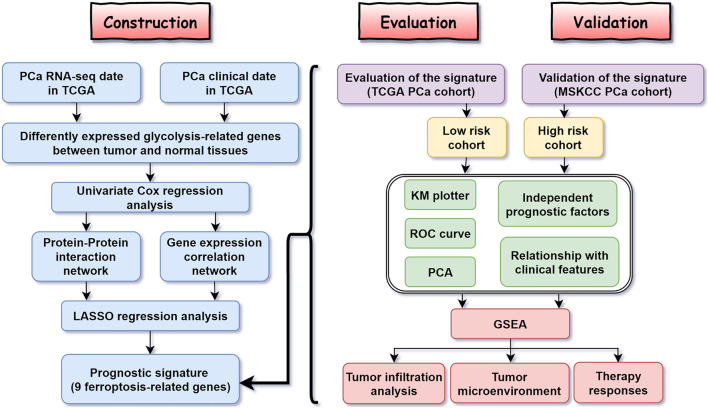
The flow diagram of the study.

**FIGURE 2:**
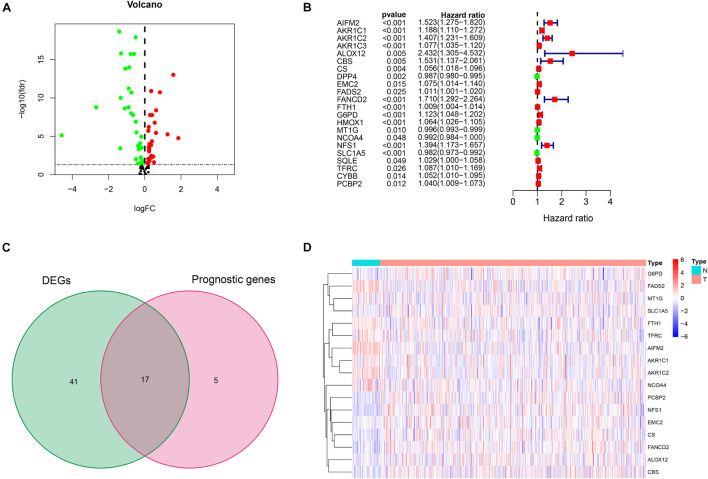
Initial screening of potential prognostic ferroptosis-related genes in The Cancer Genome Atlas (TCGA) cohort. **(A)** Volcano plot of differential expression of ferroptosis-related genes between prostate cancer (PCa) tissues and normal tissues. **(B)** Twenty-two ferroptosis-related genes associated with biochemical recurrence (BCR) of PCa patients through univariate Cox regression analysis. **(C)** Identification of 17 prognostic genes by intersection. **(D)** Heat map of the expression of 17 prognostic genes between PCa and normal tissues.

Figure 1 shows the flow diagram of this study. Among 84 ferroptosis-related genes, 31 were underexpressed, and 27 were highly expressed in tumor tissues compared with the corresponding normal tissues in the TCGA cohort (FDR < 0.05) (Figure 2A and Supplementary Table 1). To identify prognostic genes associated with the bRFS, the univariate Cox regression analysis suggested that 22 ferroptosis-related genes correlated with the prognosis of PCa (Supplementary Table 2). Only four genes serve as protective factors, while the other 18 genes were risk factors (Figure 2B). Taking the intersection of the two lists of genes, 17 ferroptosis-related genes were identified as candidate genes for the subsequent construction of a prognostic model (Figure 2C). The expression of these genes in tumor tissues and adjacent normal tissues is shown in Figure 2D.

**FIGURE 3:**
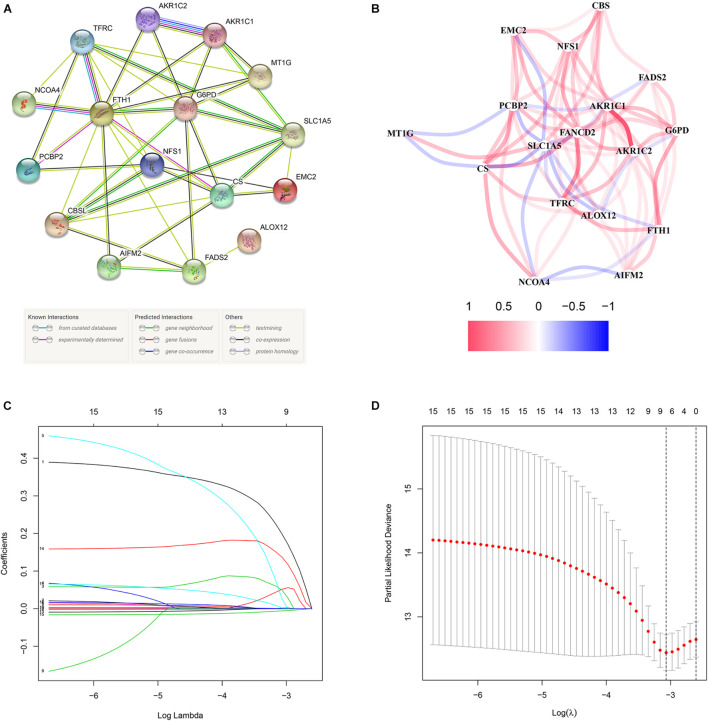
The network of candidate genes and construction of the signature in TCGA cohort. **(A)** The construction of protein–protein interaction (PPI) network by STRING. **(B)** The gene expression correlation network. **(C,D)** A nine-mRNA signature was constructed by LASSO Cox regression.

These 17 bRFS-related genes were uploaded to STRING to construct a protein–protein interaction (PPI) network (Figure 3A). The correlation analysis also showed that these genes were strongly correlated at the transcriptional level (Figure 3B). To screen the colinearity of these 17 genes, the LASSO Cox regression analysis was performed to determine the real bRFS-affecting factors and finally identified a prognostic panel of 9 ferroptosis-related genes (Figures 3C,D). The following formula was used to calculate the risk assessment score: risk score = *AIFM2* × (0.19867) + *AKR1C1* × (0.05148) + *AKR1C2* × (0.04941) + *CBS* × (0.02658) + *FANCD2* × (0.00202) + *FTH1* × (0.00027) + *G6PD* × (0.00189) + *NFS1* × (0.13708) + *SLC1A5* × (-0.00602).

### Evaluation and Validation of the Prognostic Signature

**FIGURE 4:**
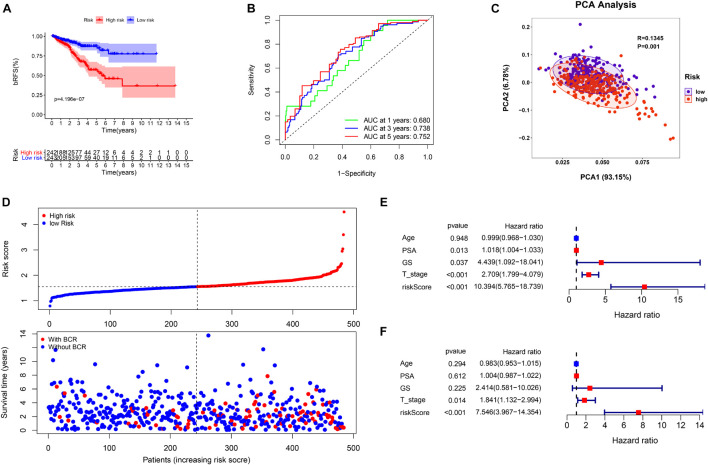
The evaluation of prognostic signature in TCGA cohort. **(A)** The biochemical relapse-free survival (bRFS) analysis of the two subgroups stratified based on the median of risk scores calculated by the risk model. **(B)** Receiver operating characteristic (ROC) curve of model and clinical characteristics predicting 1-, 3-, and 5-year bRFS. **(C)** The two risk groups were distinguished by principal component analysis (PCA). **(D)** The curve of risk score and BCR status of the patients. **(E,F)** Univariate and multivariate Cox regression analyses showed that the risk score had prominent prognostic values.

Based on the signature calculation formula, all patients in the TCGA cohort were divided into high- and low-risk groups according to their median risk score. The K-M curve showed that the high-risk group had a higher probability of BCR (*p* < 0.001) (Figure 4A). When evaluating bRFS prediction, the 1-, 3-, and 5-year AUCs of the developed gene signature were 0.680, 0.738, and 0.767, respectively (Figure 4B). PCA demonstrated that patients in various risk groups showed different two-dimensional spatial distributions (*p* < 0.001) (Figure 4C). When all patients were ranked according to their risk scores, the proportion of patients with BCR in the high-risk group was significantly higher than that in the low-risk group (Figure 4D). Univariate and multivariate Cox regression analyses were implemented to evaluate whether the model was an independent predictor among other clinical factors such as age, prostate-specific antigen (PSA), Gleason score (GS), and staging. We found that the risk score remained independently associated with bRFS not only at the univariate but also at the multivariate analysis when combined with all the clinical features (*p* < 0.05) (Figures 4E,F and Supplementary Table 3).

**FIGURE 5:**
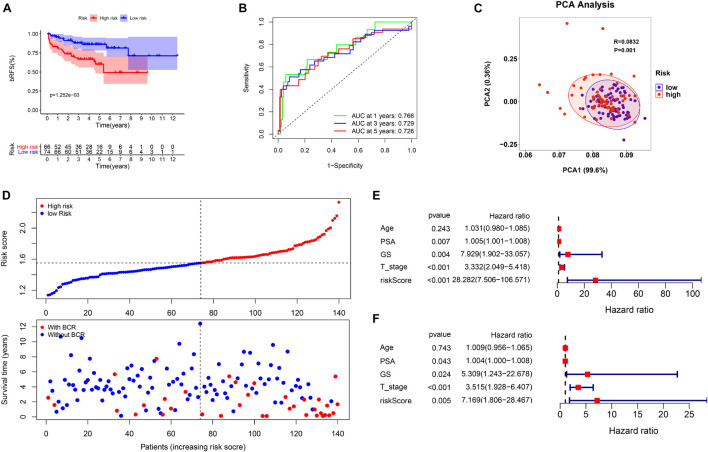
The validation of prognostic signature in MSK-IMPACT Clinical Sequencing Cohort (MSKCC) cohort. **(A)** The bRFS analysis of the two subgroups stratified based on the median of risk scores calculated by the risk model. **(B)** ROC curve of model and clinical characteristics predicting 1-, 3-, and 5-year bRFS. **(C)** The two risk groups were distinguished by PCA. **(D)** The curve of risk score and BCR status of the patients. **(E,F)** Univariate and multivariate Cox regression analyses showed that the risk score had prominent prognostic values.

This study evaluated the MSKCC to verify the predictive robustness of this model. To avoid false positives caused by information bias, the same statistical methods were used. Surprisingly, the validation results were highly consistent with that in the TCGA cohort. Survival analysis also demonstrated that a high-risk score was associated with the poor bRFS of patients with PCa (Figure 5A). The AUC values for the prognostic model for bRFS were 0.766 at 1 year, 0.729 at 3 years, and 0.726 at 5 years (Figure 5B). The PCA results also suggested that different risk subgroups showed significant discrete tendency directly in the two-dimensional plane (Figure 5C). The BCR rate in the high-risk group was significantly higher than that in the low-risk group (Figure 5D). The risk score was still an independent prognostic factor in the MSKCC (Figures 5E,F and Supplementary Table 3).

### Correlation Between Prognostic Risk Signature and Clinical Features

**FIGURE 6:**
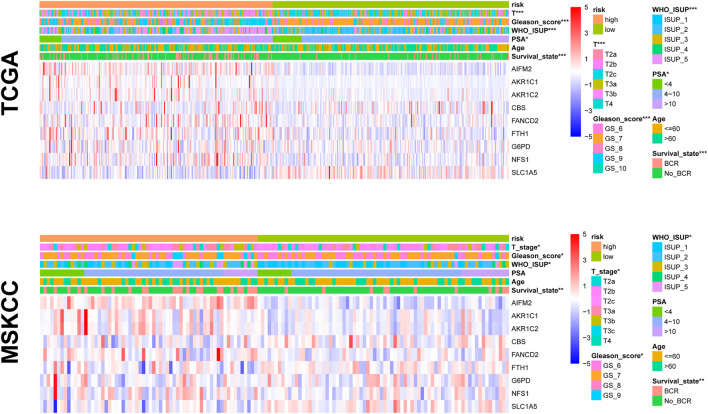
The relationship between prognosis and clinicopathological parameters in two cohorts (*p < 0.05; **p < 0.01; ***p < 0.001).

To test the predictive power of the prognostic risk model for clinical features, we correlated the risk score with the clinical features (i.e., age, BCR state, PSA, GS, WHO ISUP classification, and T-staging). The risk grouping system showed a significant correlation with the BCR state, GS, WHO ISUP classification, and T-staging but not with age and PSA in both cohorts. The high-risk group showed a higher percentage of BCR and higher WHO ISUP classification, GS, and T-staging than the low-risk group. The heat map of gene expression showed that risk factors *AIFM2*, *AKR1C1*, *AKR1C*, *CBS*, *FANCD2*, *FTH1*, *G6PD*, and *NFS1* were highly expressed in the high-risk group, while only *SLC1A5*, as a protective factor, was highly expressed in the low-risk group in both cohorts (Figure 6 and Supplementary Table 4).

### Exploration of Potential Mechanism

**FIGURE 7:**
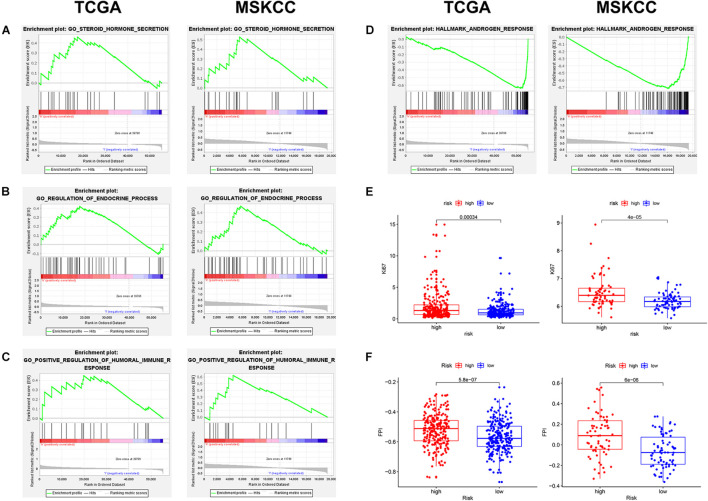
The exploration of potential mechanism about the prognostic signature in two cohorts. **(A–D)** Gene set enrichment analysis (GSEA) results of the high- and low-risk groups. **(E)** The Ki67 expression level was higher in high-risk patients. **(F)** The ferroptosis potential index (FPI) level was higher in high-risk patients.

To explore the mechanism of this risk model, GSEA was used to analyze the potential biological processes and pathways. GSEA results showed that “steroid hormone secretion,” “regulation of endocrine processes,” and “positive regulation of humoral immune response” were significantly enriched in the high-risk group (Figures 7A–C), while the low-risk group was significantly enriched in “androgen response” (Figure 7D). In addition, the levels of Ki67 expression were compared between the high- and low-risk groups, which revealed that the level of Ki67 expression was higher in the high-risk group (Figure 7E). The FPI model constructed by Liu et al. (2020c) showed that a high FPI predicts poor prognosis in various tumors, and it was associated with many important metastasis and immune-related pathways. Therefore, the relationship between the risk score of our prognostic model and FPI was explored. Similarly, the FPI of the high-risk group was significantly higher than that of the low-risk group (Figure 7F). Notably, all the presented results are consistent in the TCGA and MSKCC cohorts (Supplementary Table 5).

### Immune Infiltration and Tumor Microenvironment

**FIGURE 8:**
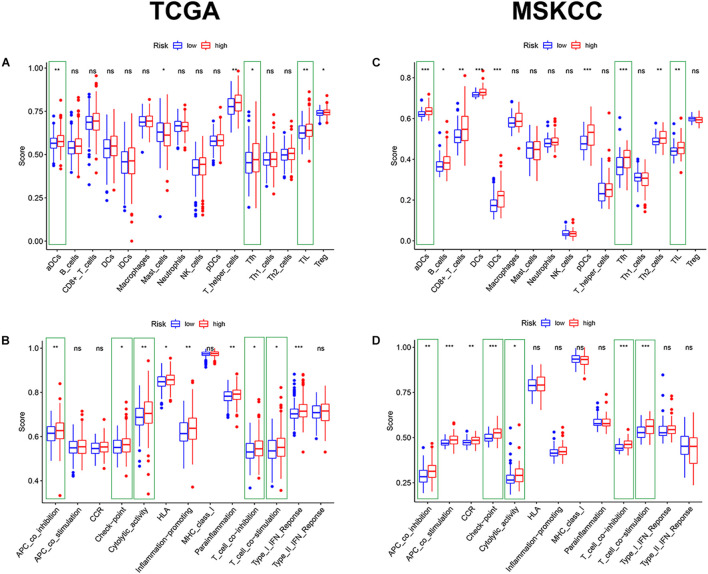
The comparison of immune cells and immune function by single-sample GSEA (ssGSEA) in the two cohorts. **(A,C)** Enrichment results of immune cell score in the two cohorts. **(B,D)** Enrichment results of immune function score in the two cohorts (*p < 0.05; **p < 0.01; ***p < 0.001).

**FIGURE 9:**
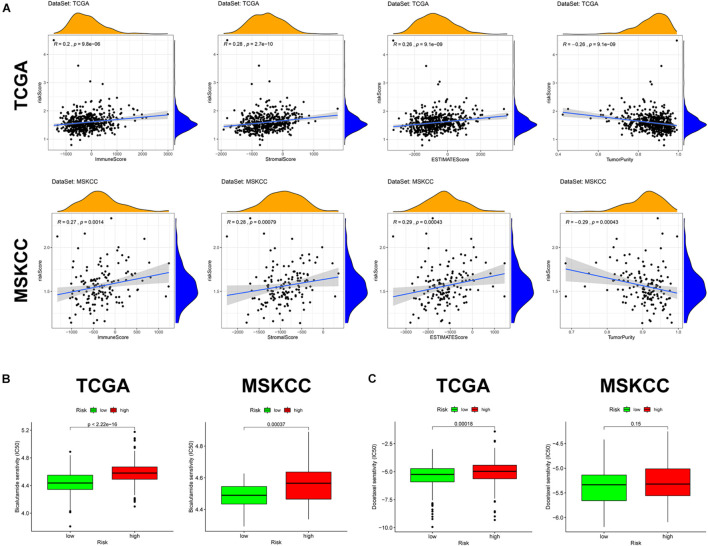
Tumor microenvironment and prediction of therapeutic response in the two cohorts. **(A)** Tumor microenvironment in the two cohorts. **(B)** Sensitivity to bicalutamide in different risk groups. **(C)** Sensitivity to docetaxel in different risk groups.

Given the above GSEA results and results of previous studies that iron death may be associated with immune cells, immune function, and immune microenvironment, we compared the differences in immune-related parameters between high- and low-risk groups in terms of the infiltration of 16 immune cells and activity of 13 immune-related pathways. In this study, most immune cell and immune function scores were higher in the high-risk group than in the low-risk group (Figure 8). In particular, aDCs, Tfh, TIL, APC co-inhibition, checkpoint, cytolytic activity, T-cell co-inhibition, and T-cell co-stimulation scores were significantly enriched in the high-risk group (Supplementary Table 6). We used the “ESTIMATE” package to evaluate TME and obtained similar results. With the increase in the risk score, the immune/stromal/ESTIM scores as a predictor of TME also increased, and the purity of the tumor decreased (Figure 9A and Supplementary Table 7).

### Prediction of Antiandrogenic Therapy and Immunotherapy Responses

Alongside radical surgery and radiotherapy, drug therapy, including endocrine therapy and chemotherapy, is an important treatment of PCa. In the GSEA, “androgen response” was significantly enriched in the low-risk group, so we explored whether patients in the high- and low-risk groups responded differently to bicalutamide. The estimated IC50 value demonstrated that the low-risk group in both cohorts had a better response to bicalutamide (*p* < 0.0001) (Figure 9B). Similarly, the level of Ki67 expression was higher in the high-risk group than in the low-risk group, suggesting that the high-risk group had more active tumor cell proliferation, faster tumor growth, and worse tissue differentiation than the low-risk group. This also raises the question on whether the response to chemotherapy is different between the high- and low-risk groups. Docetaxel, the most classic chemotherapy drug for PCa, was also found to have a better response in the low-risk group of the two cohorts, but the difference was not significant in the MSKCC cohort (*p* = 0.15) (Figure 9C).

### Nomogram for Biochemical Relapse-Free Survival Prediction

**FIGURE 10:**
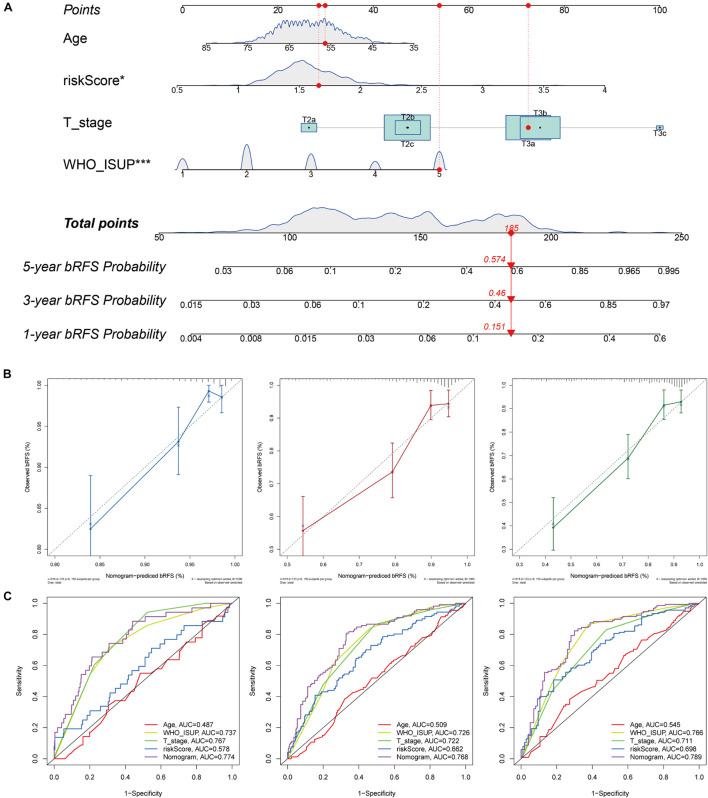
Nomogram for bRFS prediction. **(A)** A prognostic nomogram including signature risk score and other clinical factors. For example, a patient with an age of 56.37, WHO ISUP of 5, a T stage of T3a, and a signature score of 1.6597 had a total nomogram score of 185, implying the 1-, 3-, and 5-year bRFS probability of 15.1, 46, and 57.4%, respectively. **(B)** The calibration curves of the 1-, 3-, and 5-year bRFS. **(C)** ROC curve used to evaluate the 1-, 3-, and 5-year bRFS predictive efficiency.

To improve the accuracy of the risk score model in predicting bRFS, we combined the risk scores of the two cohorts and constructed a nomogram with conventional clinicopathological features (Figure 10A). The C-index (0.751) showed good predictive accuracy for the nomogram. The calibration curves had good linearity for the 1-, 3-, and 5-year bRFS (Figure 10B). The nomogram scores achieved a higher AUC–ROC than all other clinicopathological parameters in predicting 1-, 3-, and 5-year bRFS (AUC at 1 year, 0.774; AUC at 3 years, 0.768; AUC at 5 years, 0.789) (Figure 10C).

### *AIFM2* and *NFS1* Were Differentially Expressed in Prostate Cancer Tissues and Were Associated With Poor Outcome

**FIGURE 11:**
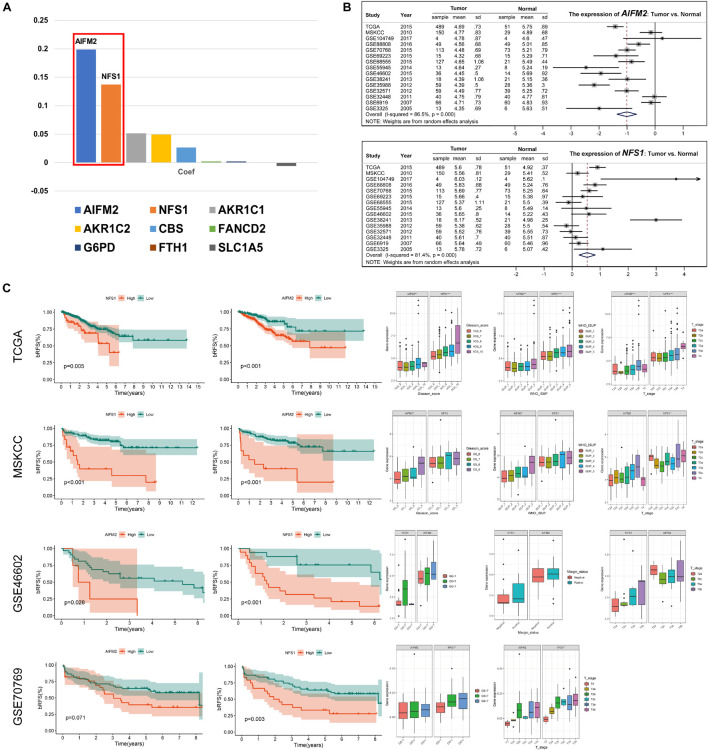
*AIFM2* and *NFS1* were differentially expressed in PCa tissues and were associated with poor outcome. **(A)** The weight coefficient of each component in the signature. **(B)** The differential expression of *AIFM2* and *NFS1* in tumor and normal tissues based on multiple datasets. **(C)** *AIFM2* and *NFS1* were significantly associated with prognosis based on multiple datasets (*p < 0.05; **p < 0.01; ***p < 0.001).

**FIGURE 12:**
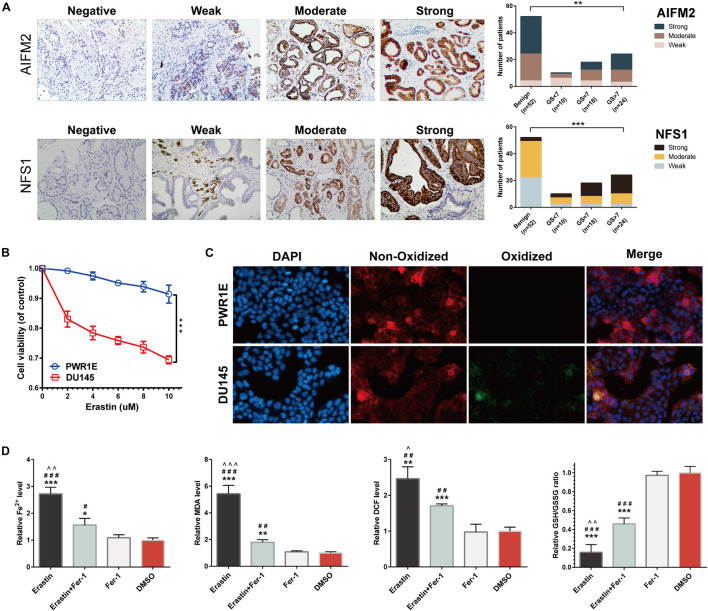
Immunohistochemistry (IHC) verification of *AIFM2* and *NFS1* and sensitivity verification of PCa cells to ferroptosis. **(A)** The protein expression of *AIFM2* and *NFS1* in tumor and normal tissues by IHC. **(B)** Cell viability was assessed following exposure of DU145 cells and PWR1E cells to different concentrations of erastin. **(C)** Immunofluorescence staining of oxidized lipid reactive oxygen species (ROS) (green color) and reduced lipid ROS (red color) formation in DU145 and PWR1E cells treated with erastin for 24 h. **(D)** Fe^2+^ release, malondialdehyde (MDA), DCF, and glutathione (GSH) levels detected by Assay Kit in DU145 cells incubated with erastin, ferrostatin-1, both, and DMSO for 24 h (*p < 0.05; **p < 0.01; ***p < 0.001; #*p* < 0.05; ##*p* < 0.01; ###*p* < 0.001; ^∧^*p* < 0.05; ^∧∧^*p* < 0.01; ^∧∧∧^*p* < 0.001).

To further explore the mechanisms involved in the risk signature, we focused on the nine ferroptosis-related genes. The coefficients of *AIFM2* and *NFS1* genes were significantly higher than those of the other seven genes involved in the construction of the signature, and a larger weight often indicates that the gene is more prognostically important (Figure 11A). Therefore, *AIFM2* and *NFS1* became the focus of the subsequent research. First, we evaluated the differential expression of *AIFM2* and *NFS1* between the tumor tissues and corresponding normal tissues in multiple datasets. The results of a meta-analysis after combining 15 independent datasets showed that the expression of *AIFM2* in the tumor was lower than that in normal tissues, while opposite results were obtained in *NFS1* (*p* < 0.001) (Figure 11B). Furthermore, we found that patients with a high expression of *AIFM2* or *NFS1* had earlier onset of biochemical relapse and that a high expression of *AIFM2* or *NFS1* was associated with higher GS and WHO ISUP grade, poorer clinicopathological stage, and higher positive rate of the surgical margin, but some of the results were not significant because of the small sample size (Figure 11C). To verify these findings, IHC of these two genes was performed in 52 pairs of tumor tissues and corresponding normal tissues after radical prostatectomy. The actual results were consistent with the expected results. The expression of *AIFM2* was lower in tumor tissues than in normal tissues, but the high expression of *AIFM2* tended to have higher GS (*p* < 0.01). *NFS1* is highly expressed in tumors; likewise, highly expressed *NFS1* tends to have higher GS (*p* < 0.001) (Figure 12A).

### Prostate Cancer Cells Are More Sensitive to Ferroptosis Than Normal Prostate Cells

We assessed the sensitivity of PCa cells (DU145) to ferroptosis. Erastin (ferroptosis inducer) treatment decreased the viability of DU145 cells in a dose-dependent manner. Compared with normal human prostate cells (PWR1E), the activities of DU145 cells were significantly decreased at any concentrations of erastin (*p* < 0.001) (Figure 12B). By using BODIPY^TM^ 581/591 C11 as a lipid peroxidation probe, we confirmed that the DU145 cell lines induced by erastin produced more lipid peroxidations (green) than the PWR1E cell lines (Figure 12C). In addition, the levels of Fe^2+^ release, MDA, DCF, and GSH in DU145 cells were measured for 24 h under different treatment conditions. After induction by erastin, Fe^2+^, MDA, and DCF levels in DU145 cells were significantly increased, while the GSH level was significantly decreased. This trend can be partially rescued by ferrostatin-1, a ferroptosis inhibitor (Figure 12D).

### *AIFM2* and *NFS1* Knockdowns Promote Ferroptosis and Suppress Proliferation *in vitro* and *in vivo*

**FIGURE 13:**
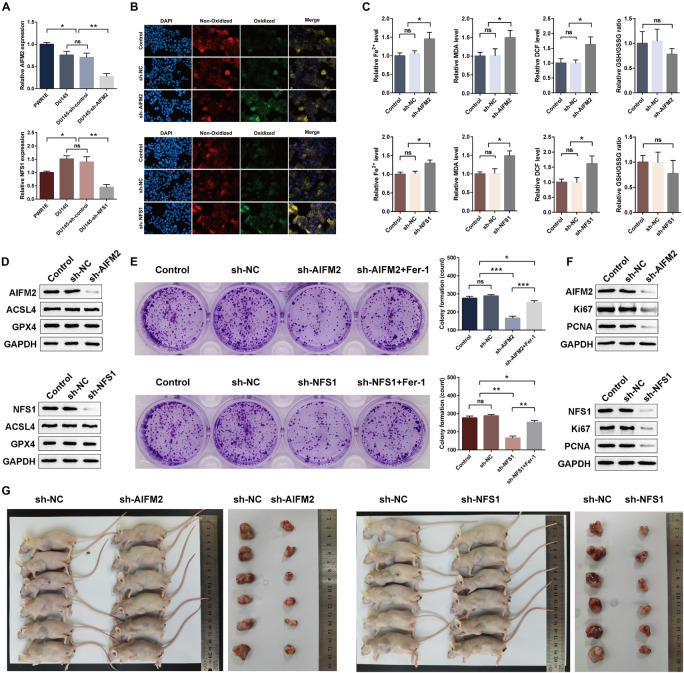
*AIFM2* and *NFS1* knockdowns promote ferroptosis and suppress proliferation *in vitro* and *in vivo*. **(A)** The real-time quantitative polymerase chain reaction (RT-qPCR) analysis of *AIFM2* and *NFS1* expression levels in PWR1E, DU145, DU145 sh-control, and DU145 sh-*AIFM2/NFS1*. **(B)** Immunofluorescence staining of oxidized lipid ROS (green color) and reduced lipid ROS (red color) formation in DU145 after *AIFM2/NFS1* knockdown. **(C)** Fe^2+^ release, MDA, DCF, and GSH levels detected by Assay Kit in DU145 cells after *AIFM2/NFS1* knockdown. **(D)** The Western blot analysis of *ACSL4* and *GPX4* expression levels after *AIFM2/NFS1* knockdown. **(E)** Colony formation assays were constructed in *AIFM2/NFS1* knockdown DU145 cells. **(F)** The Western blot analysis of *Ki67* and *PCNA* expression levels after AIFM2/NFS1 knockdown. **(G)** Xenograft tumors in nude mouse models (*p < 0.05; **p < 0.01; ***p < 0.001).

Moreover, we detected a difference in the expressions of the *AIFM2* or *NFS1* genes between PCa cell lines and normal cell lines, which was consistent with the analysis results of the tissue samples (Figure 13A). To evaluate the potential role of *AIFM2* and *NFS1* in the regulation of PCa ferroptosis, human PCa DU145 cells were treated with control shRNA or target genes shRNA (Figure 13A). Erastin-induced lipid peroxidation measured with BODIPY^TM^ 581/591 C11 could be promoted by sh-AIFM2 and sh-NFS1 (Figure 13B). As expected, *AIFM2* and *NFS1* knockdowns by shRNA notably increased the levels of Fe^2+^, MDA, and DCF, but decreased the level of GSH (but not significantly) (Figure 13C). The above results indicated that *AIFM2* or *NFS1* knockdown could promote the ferroptosis of PCa cell lines. To date, the phospholipid hydroperoxide-reducing enzyme glutathione peroxidase 4 (*GPX4*) has been known to be the main enzyme that protects against ferroptosis (Stockwell et al., 2017), and Acyl-CoA synthetase long-chain family member 4 (*ACSL4*) is also a key contributor and regulator of ferroptosis, which determines the sensitivity of ferroptosis (Yuan et al., 2016; Doll et al., 2017). Therefore, we tried to explore whether the *AIFM2* and *NFS1* knockdowns affect ferroptosis by affecting the expressions of *GPX4* and *ACSL4* proteins. However, we did not observe significantly positive results (Figure 13D), which suggests the involvement of other pathways. In addition, colony formation assays indicated that *AIFM2* or *NFS1* knockdown significantly inhibited cell colony formation. However, it is unclear whether the reduced clonogenic capacity is due to the increase in ferroptosis sensitivity or the decrease in tumor cell proliferation. Therefore, ferrostatin-1 was added after *NFS1*/*AIFM2* knockdown to inhibit ferroptosis. The addition of ferrostatin-1 partially rescued the reduced clonogenic capacity in both sh-*AIFM2* and sh-*NFS1* cells (Figure 13E). This illustrated the decreased ability to form colonies after *NFS1*/*AIFM2* knockdown, on the one hand, by promoting ferroptosis, and on the other, by inhibiting cell proliferation ability. This was indeed confirmed by subsequent detection of proliferation markers *Ki67* and proliferating cell nuclear antigen (*PCNA*), which were significantly downregulated in both sh-*AIFM2* and sh-*NFS1* cells (Figure 13F). Finally, we further explored the effects of *AIFM2* and *NFS1* on the development of PCa *in vivo*. We found that *AIFM2* and *NFS1* knockdowns both suppressed PCa cell tumorigenicity (Figure 13G).

## Discussion

Ferroptosis, as a newly discovered form of RCD, regulates cells to death by accumulating iron-dependent extensive lipid peroxidation. This concept was first proposed by Dixon et al. (2012), and its research has grown exponentially in the past few years. Although ferroptosis is still a mysterious veil in physiology, its role in the human pathological state, especially in cancer, has been extensively studied (Jiang et al., 2021). Many studies have recently identified ferroptosis as a natural mechanism of tumor inhibition and have shown that inactivation of ferroptosis can promote tumor development, just like inactivation of apoptosis (Jiang et al., 2015; Zhang et al., 2018). Ferroptosis is also important in the systematic treatment, radiotherapy, and immunotherapy of cancer (Chen et al., 2021). Therapies that rely on ferroptosis provided a new field of cancer treatment (Conrad et al., 2021). Therefore, ferroptosis may become a new marker and a potential prognostic indicator of malignant tumors.

As we suspected, ferroptosis and its related gene signature are closely related to the prognosis of various cancers. Liu et al. (2020a) and Zhuo et al. (2020) constructed ferroptosis-related gene signatures, which were significantly correlated with the diagnosis and prognosis of gliomas. Liang et al. (2020) and Liu et al. (2020b) also developed novel gene signatures associated with ferroptosis, which can be used to predict the prognosis of hepatocellular carcinoma. Tang R. et al. (2020) reported that the ferroptosis pathway is mainly involved in the prognosis of pancreatic cancer and proposed that the combination of immunotherapy and chemotherapy with ferroptosis inducer may be a feasible treatment of pancreatic cancer. Similar research results can also be found in lung adenocarcinoma, ovarian carcinoma, and uveal melanoma (Gao et al., 2021; Luo and Ma, 2021; Yang L. et al., 2021). However, no studies have attempted to construct a ferroptosis-related prognostic model of PCa. PCa mainly relies on lipid metabolism to obtain energy (Liu, 2006). The overexpression of lipid metabolism-related genes and proteins has been found in its early and late stages, even in metastatic lesions (Swinnen et al., 2002; Ettinger et al., 2004; Chen et al., 2018; Iglesias-Gato et al., 2018; Zadra and Loda, 2018). These observations suggest that PCa, as a lipid metabolic tumor, may be sensitive to ferroptosis. Ghoochani et al. (2021) used erastin and RSL3, which are ferroptosis inducers, to significantly reduce the growth and migration of PCa cells *in vitro* and significantly delayed the growth of drug-resistant prostate tumors *in vivo* without noticeable side effects. Similarly, Zhou et al. (2020) used flubendazole to elicit valid antitumor effects by promoting ferroptosis in CRPC. Their results are sufficient to confirm that PCa is closely related to ferroptosis, which provides an opportunity to develop a ferroptosis-related prognosis model.

High-throughput gene sequencing technology for biological samples enables large-scale omics research. In this study, we tried to explore a genetic marker based on ferroptosis to predict the BCR of PCa. First, we obtained 17 ferroptosis-related genes potentially associated with the BCR of PCa based on the difference analysis between tumor tissue and normal tissue and results of the univariate Cox regression analysis. Both the protein interaction and gene expression correlation networks suggested that these 17 genes had significant functional and expression correlations. Thus, based on these 17 genes, Lasso regression analysis was adopted to finally construct a nine-ferroptosis-related gene prognosis signature. bRFS analysis showed that the signature could strongly predict the BCR of PCa. Univariate and multivariate Cox regression analyses showed that the calculated risk score was an independent risk factor for the BCR of PCa. In addition, the risk score was positively correlated with poor clinicopathological features, including BCR state, GS, WHO ISUP classification, and T-staging. Mechanism exploration results showed that a high-risk score was associated with steroid secretion, endocrine process, and humoral immune response, while a low-risk score was associated with androgen response. Metabolic recombination and immune evasion are two distinct characteristics of cancer, but recent studies have shown a close relationship between them (Chang et al., 2015; Hao et al., 2019). The metabolic competition between tumor and immune cells may lead to tumor immunosuppression (Chang et al., 2015). We confirmed that the higher the risk score, the higher the degree of immune cell infiltration, the more active the immune-related function, and the higher the immune/stromal/ESTIM scores. Owing to the different expressions of Ki67 and different responses to androgen in high- and low-risk groups, we explored whether the signature could predict the response of patients to chemotherapy and endocrine therapy. Predictably, patients in the low-risk group responded better to docetaxel and bicalutamide than those in the high-risk group. Notably, all the above results were confirmed in two independent PCa cohorts (TCGA and MSKCC).

In our analysis of the nine-gene model, among the most important predictors of the model, we found that *AIFM2* and *NFS1* genes account for more than half of the weight of the model, which mainly determines the risk score of the patients. In our review of existing studies, *AIFM2*, also called ferroptosis suppressor protein 1, can catalyze CoQ10 regeneration by NAD(P)H. As a lipophilic radical-trapping antioxidant, CoQ10 can halt the propagation of lipid peroxides and prevent the damage of the plasma membrane from peroxide. *AIFM2* protects tumor cells by catalyzing the continuous regeneration of CoQ10 and improving the ability to trap lipid peroxyl radicals to inhibit ferroptosis. The above findings were completed almost simultaneously by two research teams and published back-to-back in the famous journal *Nature* (Bersuker et al., 2019; Doll et al., 2019). Alvarez et al. (2017) found that *NFS1* was generally highly expressed in lung cancer tissues and cell lines. Further experiments showed that *NSF1* collected more sulfur elements from cysteine to produce iron–sulfur clusters, which reduced the release of iron from cells and significantly alleviated hyperoxia-induced ferroptosis. In animal experiments, the tumor formation time was significantly prolonged after *NSF1* knockdown, suggesting a positive selection for *NSF1* during the development of lung cancer to overcome cell ferroptosis. These findings were also published in *Nature*. Subsequently, we conducted *in vivo* and *in vitro* experiments to evaluate the prognostic significance of these two genes in PCa and to explore their influence on ferroptosis, and the results were widely validated.

In addition to *AIFM2* and *NFS1*, the remaining seven genes in the signature were also associated with cancer in both basic and clinical research fields. *AKR1C1* and *AKR1C2* were prognostic factors of breast cancer (Wenners et al., 2016), and selective loss of these genes may help enhance endocrine therapy in breast cancer (Ji et al., 2004). Moreover, the overexpression of *AKR1C1/AKR1C2* may serve as a biomarker of chemoresistance in non-small cell lung cancer cells (Wang et al., 2007). Recent studies have shown that CBS promotes the growth of colon and ovarian cancer in preclinical models (Zhu et al., 2018), and CBS blockers have the potential as adjuvants in the treatment of breast cancer to reduce the ability of cancer cells to resist oxidative stress induced by many chemotherapeutic drugs (Sen et al., 2015). *FANCD2* was a sensitive and independent prognostic factor for breast cancer (Fagerholm et al., 2013), and its overexpression is a reliable indicator of lymph node metastasis in colorectal cancer (Ozawa et al., 2010). *FTH1* was highly expressed in primary liver tumors, and its lower expression was associated with better survival (Muhammad et al., 2020). FTH1 pseudogenes, as competitive endogenous RNAs, play various roles in oncology, particularly in PCa (Chan et al., 2018; Di Sanzo et al., 2020). Blocking the glycosylation of G6PD, the rate-limiting enzyme of the pentose phosphate pathway, can reduce the proliferation of cancer cells *in vitro* and tumor growth *in vivo* (Rao et al., 2015). *SLC1A5* plays an important role in glutamine transport by controlling the metabolism, growth, and survival of lung cancer cells (Hassanein et al., 2013), and its variant is a mitochondrial glutamine transporter for cancer metabolic reprogramming (Yoo et al., 2020).

This study has some limitations. First, the results were based on retrospective public datasets that need to be verified in a prospective cohort in the future. Second, owing to the inherent intratumoral heterogeneity and the technical noise caused by cross-platform sequencing, how to correctly standardize the expression data is the ultimate challenge for the clinical application of this nine-gene signature. Therefore, the RNA-seq data of the patients need data preprocessing, such as scaling and normalization, in future clinical applications. It is even possible to develop a standardized and commercial gene detection kit based on these nine genes, which can automatically calculate the risk score for risk grouping. Finally, the underlying biological mechanisms of this signature, in particular, how *AIFM2* and *NFS1* influence the ferroptosis process in PCa, remain unknown. At present, research on the two genes is still in the embryonic stage, so further research is needed.

In summary, we constructed a nine-gene signature associated with ferroptosis, which can accurately predict the BCR of PCa. The higher the risk score, the higher the probability of developing BCR, the worse the clinicopathological characteristics, and the worse the response to chemotherapy and antiandrogen therapy. This signature can be used as a novel tool for distinguishing high- and low-risk PCa populations and help in understanding the mechanism of cellular ferroptosis in the carcinogenesis and development of PCa.

## Data Availability Statement

Publicly available datasets were analyzed in this study. This data can be found here: All raw data can be downloaded from the TCGA Research Network (https://www.cancer.gov/tcga), Cbioportal database (https://www.cbioportal.org/), Gene Expression Omnibus repository (https://www.ncbi.nlm.nih.gov/geo/), and the MSigDB (https://www.gsea-msigdb.org/gsea/msigdb/).

## Ethics Statement

The studies involving human participants were reviewed and approved by the Ethics Committee of the Beijing Hospital. The patients/participants provided their written informed consent to participate in this study. The animal study was reviewed and approved by the Institutional Ethics Committee of Xiangya Hospital, Central South University.

## Author Contributions

ZL developed the methodology, performed the formal analysis, and wrote the original draft. JLW supervised the study and acquired the funding. XW was in charge of the data curation and acquired the funding. MM acquired the funding. GT was in charge of the software and validation. HX investigated and validated the study. JYW was in charge of the project administration. YL acquired the funding and resources. ML acquired the funding and wrote, reviewed, and edited the manuscript. All authors contributed to the article and approved the submitted version.

## Conflict of Interest

The authors declare that the research was conducted in the absence of any commercial or financial relationships that could be construed as a potential conflict of interest.

## Publisher’s Note

All claims expressed in this article are solely those of the authors and do not necessarily represent those of their affiliated organizations, or those of the publisher, the editors and the reviewers. Any product that may be evaluated in this article, or claim that may be made by its manufacturer, is not guaranteed or endorsed by the publisher.

## Footnotes

1
https://portal.gdc.cancer.gov/

2 https://www.cbioportal.org/, or GSE21032.

3
https://www.ncbi.nlm.nih.gov/geo/

4
https://www.gsea-msigdb.org/gsea/msigdb/

5
https://string-db.org/

6
https://www.cancerrxgene.org
